# A systematic review of hospital accreditation: the challenges of measuring complex intervention effects

**DOI:** 10.1186/s12913-015-0933-x

**Published:** 2015-07-23

**Authors:** Kirsten Brubakk, Gunn E. Vist, Geir Bukholm, Paul Barach, Ole Tjomsland

**Affiliations:** South-Eastern Norway Regional Health Authority, Hamar, Norway; Prevention, Health promotion and Organization Unit, Norwegian Knowledge Centre for the Healthcare Services, Oslo, Norway; Norwegian Institute of Public Health, Oslo, Norway; Wayne State University School of Medicine, Michigan, USA; Department of Medicine and Health, South-Eastern Norway Regional Health Authority, Hamar, Norway

**Keywords:** Accreditation, Certification, Hospital, Patient Safety, Evaluation

## Abstract

**Background:**

The increased international focus on improving patient outcomes, safety and quality of care has led stakeholders, policy makers and healthcare provider organizations to adopt standardized processes for evaluating healthcare organizations. Accreditation and certification have been proposed as interventions to support patient safety and high quality healthcare. Guidelines recommend accreditation but are cautious about the evidence, judged as inconclusive. The push for accreditation continues despite sparse evidence to support its efficiency or effectiveness.

**Methods:**

We searched MEDLINE, EMBASE and The Cochrane Library using Medical Subject Headings (MeSH) indexes and keyword searches in any language. Studies were assessed using the Cochrane Risk of Bias Tool and AMSTAR framework. 915 abstracts were screened and 20 papers were reviewed in full in January 2013. Inclusion criteria included studies addressing the effect of hospital accreditation and certification using systematic reviews, randomized controlled trials, observational studies with a control group, or interrupted time series. Outcomes included both clinical outcomes and process measures. An updated literature search in July 2014 identified no new studies.

**Results:**

The literature review uncovered three systematic reviews and one randomized controlled trial. The lone study assessed the effects of accreditation on hospital outcomes and reported inconsistent results. Excluded studies were reviewed and their findings summarized.

**Conclusion:**

Accreditation continues to grow internationally but due to scant evidence, no conclusions could be reached to support its effectiveness. Our review did not find evidence to support accreditation and certification of hospitals being linked to measurable changes in quality of care as measured by quality metrics and standards. Most studies did not report intervention context, implementation, or cost. This might reflect the challenges in assessing complex, heterogeneous interventions such as accreditation and certification. It is also may be magnified by the impact of how accreditation is managed and executed, and the varied financial and organizational healthcare constraints. The strategies hospitals should impelment to improve patient safety and organizational outcomes related to accreditation and certification components remains unclear.

**Electronic supplementary material:**

The online version of this article (doi:10.1186/s12913-015-0933-x) contains supplementary material, which is available to authorized users.

## Background

Patient safety and patient centered care are emerging as key drivers in healthcare reform.

Accreditation is the most frequently external quality assessment of healthcare organizations’ strategic goals [1]. We defined hospital accreditation programs as the systematic assessment of hospitals against accepted standards [2] and certification is a confirmation of characteristics of an object, person, or organization against published standards [3]. Little information is available on effective accreditation and certification strategies. Prominent national organizations have recommended accreditation which is being implemented widely. However, little evidence supports their effect on patient outcomes or other important markers such as core measures, organizational culture nor reliability.

Hospital accreditation was started by The American College of Surgeons 100 years ago, and since then the number of hospital accreditation programs has expanded rapidly. The World Health Organization identified 36 nationwide healthcare accreditation programs in 2000 [4]. Accreditation is an essential part of healthcare systems in more than 70 countries and is often provided by external and independent review, assessment or audit [5]. The systematic evaluation of healthcare services is a way to obtain regulatory peer review on the organizational maturity and reliability [6]. Literature reviews on the effects of accreditation on the quality of care do not provide strong evidence due to limitations of the studies [7–12].

Greenfield and Braithwaite [7] identified the effects of accreditation on promoting change and professional development, indicating that the effects were probably due to accreditation and certification, but lacking firm evidence. A systematic review by Nicklin *et al.* [8] found several positive benefits of accreditation, however, the study lacked rigor to support their conclusions. Shaw *et al.* [13] found evidence for positive effects between accreditation, certification and clinical leadership, systems for patient safety and clinical review, but was fell short of endorsing accreditation, and concluded with recommending further analysis to explore the association of accreditation and certification with clinical outcomes. Furthermore, Ho *et al.* have demonstrated an unintended negative impact on the learning environment of medical students and trainees, including decreased clinical learning opportunities, increased non-clinical workload, and violation of professional integrity in preparation and during accreditation and certification [14].

The aim of this study is to systematically assess the effects of accreditation and/or certification of hospitals on both organizational processes and outcomes.

## Methods

We searched for published articles that assessed the effects of accreditation and/or certification of hospitals. The studies were reviewed for their research design and internal validity. We assessed each study’s findings in regard to their effects on patient mortality, morbidity, patient safety, as well as process outcomes.

### Data sources and search strategy

We searched MEDLINE, EMBASE, CRD, and the Cochrane Library, including the Cochrane Database of Systematic Reviews (CDSR), Database of Abstracts of Reviews of Effects (DARE) and Health Technology Assessment Database (HTA) for all studies on accreditation/certification in 2006 [11], and this was repeated in 2009 [12], 2013 and 2014. The same search criteria were used to monitor the studies addressing effect of accreditation/certification in hospitals.

The search was designed and conducted by an information specialist librarian who updated the search strategies from 2006 to 2009 and used the combinations of key words and Medical Subject Heading terms (MeSH) related to accreditation, certification and hospitals. The reference lists of selected articles were searched for potentially relevant studies meeting the inclusion criteria (snowballing). In addition, we used Google search engine using the search words accreditation, certification and patient safety. We updated our literature search in July 2014, searching the same databases with the same inclusion criteria. We found no relevant additional studies to include in our analysis.

### Study selection

We included studies identified in any language using the search strategy with the following study design: systematic reviews, randomized controlled trials (RCT), non-randomized controlled trials, controlled before and after studies (CBAs), and interrupted time series (ITS) (defined as at least three measurements before and three after the introduction of accreditation and/or certification).

The inclusion criteria used were:

- Population: all types of hospitals were included.
- Intervention: all types of accreditation and/or certification of hospitals.
- Comparison: any hospital that was not accredited or certified, either by not seeking or not receiving accreditation and/or certification.
- Outcomes: both clinical outcomes and process measures.

Two of the authors (GEV, KB) independently reviewed all titles, references and abstracts generated by the original search in order to identify articles for potential inclusion. All reports, independent of language, were evaluated for the inclusion criteria.

Each article considered potentially eligible according to the chosen criteria was independently read in full text and then assessed using a standardized form for internal validity by two authors (GEV, KB,). If several estimates for one study outcome were reported, the most fully adjusted estimate was abstracted. Each assessment was conducted independently by two reviewers, the results were compared, and the differences were all reconciled by consensus.

This study did not involve human material or human data, so an ethic approval was not needed. No written consent was obtained from participants for this literature study. Additional file 1 provides a complete description of the search strategies; and Additional file 2 provides a detailed overview of the updated search results. The PRISMA checklist (Preferred Items for Systematic Reviews and Meta-Analyses) was used for this systematic review. Please see Additional file 3.

## Results

### Search results

Our search of electronic databases identified a considerable increase in studies addressing the effect of accreditation and/or certification. In 2006, 672 studies were identified [11]. Over the next 3 years 522 new studies were published. In 2013 we identified another 910 relevant studies. Of the identified studies in 2013 fifteen citations were considered potentially eligible based on the inclusion criteria. Two additional articles were identified, and an additional three references were identified by manually searching the articles’ reference lists. Twenty references were considered potentially eligible and were retrieved for a full text assessment. Of these, 16 articles were excluded because they did not fulfil the inclusion criteria; Table 4 presents the excluded studies and the detailed reasons for their exclusion.

**Fig. 1:**
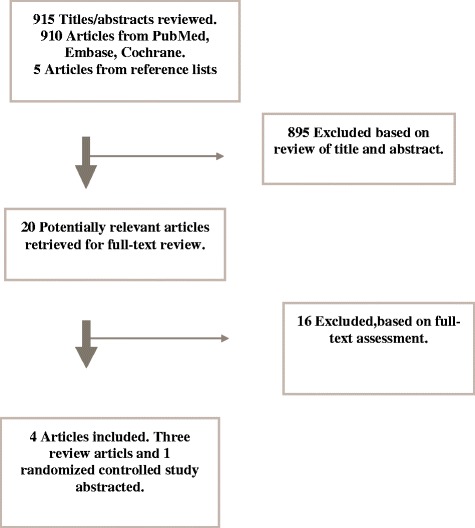
Flowchart. Flowchart of study selection process. Database searched January 18, 2013

The agreement between reviewers for study eligibility was complete. As only one original study was included a meta-analysis was not possible (Fig. 1).

### Characteristics of included studies

We included systematic reviews as well as controlled studies. A total of four references, three systematic reviews and one primary study met the inclusion [15–18]. The aims and the inclusion criteria of the three reviews were slightly different. However, their inclusion criteria overlapped with the inclusion criteria for this review. Please see Table 1 for included systematic reviews in this review.

**Table 1:** Included systematic reviews

Reference	Search date	Aim of review	Study design included	Number of included studies	Main conclusion stated by authors	Studies that match our inclusion criteria
Flodgren *et al.* 2011 [15]	May 2011	Evaluate the effectiveness of external inspection of compliance with standards in improving healthcare organizations behavior, healthcare professionals behavior and patient outcomes	RCT, CCT, ITS, CBA	Two in total, 1 RCT, 1 ITS	No firm conclusion were drawn due to paucity of high-quality controlled evaluations	Salmon *et al.* [18]
Matrix Knowledge group 2010 [16]	August 2010	Produce a general overview of results obtained and methodologies used to assess impact of accreditation	Studies containing an element of comparison	56 in total, 40 studies with quantitative design of which 1 study presented empirical data	Most studies suggest that accreditation/certification has an impact on the organization or on the professional practice. The impact on health outcomes or improvement in these outcomes was not demonstrated.	Salmon *et al.* [18]
Alkhenizan & Shaw 2011 [17]	June 2009	Evaluate the impact of accreditation programs on the quality of healthcare services	Clinical trials, observational studies and qualitative studies	26 in total, 10 studied accreditation of hospitals of which 1 had a hospital control group	Accreditation improves the process of care provided by healthcare services	Salmon *et al.* [18]

A synthesis of the three included systematic reviews

The qualities of the systematic reviews were assessed using the AMSTAR quality checklist framework, the standard for assessing methodological quality of systematic reviews [19]. The results of the assessment are shown in Table 2. Two of the reviews were of moderate quality scoring 6/11 [17], and 7/11 [16], respectively, whereas the third review was scored as high quality with a score of 9/11 [15]. Our review scored 9/11 in the AMSTAR assessment. The primary study [18] was assessed as having a high risk of bias after using the risk of bias assessment as described in the Cochrane Handbook for randomized controlled trails [20]. The assessment is shown in Table 3.

**Table 2:** AMSTAR, assessing methodological quality of systematic reviews

Study	Alkhenzian *et al.* 2011	Matrix group 2010	Flodgren *et al.* 2011	Brubakk *et al.*
AMSTAR question	Yes, No, Can’t answer, Not applicable			
1. Was an ‘a priori’ design provided?	Yes	Yes	Yes	Yes
2. Was there duplicate study selection and data extraction?	No	No	Yes	Yes
3. Was a comprehensive literature search performed?	Yes	Yes	Yes	Yes
4. Was the status of publication (i.e. grey literature) used in the inclusion criterion?	No	Yes	No	No
5. Was a list of studies (included and excluded) provided?	Yes, although only for the included	Yes, although only for the included	Yes, both included and excluded studies	Yes, both included and excluded studies
6. Were the characteristics of the included studies provided?	Yes	Yes	Yes	Yes
7. Was the scientific quality of the included studies assessed and documented?	Yes	Yes, No	Yes	Yes
8. Was the scientific quality of the included studies used appropriately in formulating conclusions?	No	Can’t answer	Yes	Yes
9. Were the methods used to combine the findings of studies appropriate?	Not applicable (N/A)	Yes	Not applicable (N/A)	Not applicable (N/A)
10. Was the likelihood of publication bias assessed?	No	No	Yes	Yes
11. Was the conflict of interest stated?	No	No	Yes	Yes

AMSTAR. Assessing Methodological Quality of Systematic Review, quality assessment of included systematic reviews categorized by yes, no, cannot answer, not applicable

**Table 3:** Risk of bias assessment of study by Salmon *et al.* [18]^a^

Domain	Support for judgement	Review author’s judgement
Selection bias		
Random sequence generation	They state stratified randomisation, but give no information about the procedure	Unclear
Allocation concealment	Not mentioned	Unclear
Performance bias		
Blinding of participants and personnel	Not mentioned and appears impossible/not possible to blind hospitals	Unclear
Detection bias		
Blinding of outcome assessor	Not mentioned	Unclear
Attrition bias		
Incomplete outcome date	The largest hospital did not complete the study. Follow- up time was shortened because controls wanted to receive the intervention	High risk
Reporting bias		
Selective reporting	Outcome selection conducted by participants and accreditor. Many outcomes/ indicators were dropped from the follow- up measurement	High risk
Other bias		
Other sources of bias	This was a cluster randomized trial, adjustment for clustering in analysis of results were not mentioned	Unclear

^a^The risk of bias assessment as described in the Cochrane Handbook for randomized controlled trails [20]

Risk of bias assessment of the included primary study by Salmon el at [18]

SOURCE: Higgins J, Green S. Cochrane Handbook for Systematic Reviews of Interventions Version 5.1.0 [updated March 2011]. The Cochrane Collaboration, 2011

### Included systematic reviews

The Cochrane review by Flodgren *et al.* has the best quality AMSTAR score [15]. The authors identified two studies which met their inclusion criteria that focused on the effect of external inspection on: a) compliance with standards improving healthcare organizations; b) healthcare professional behavior; and, c) patient outcomes.

The first study was a cluster-randomized controlled trial by Salmon *et al.* [18] that involved 20 South African public hospitals. The other study was an interrupted time- series conducted to identify the effects of the NHS Healthcare Commissions Infection Inspection program on the MRSA rates in UK trusts hospitals, but did not meet our inclusion criteria. Flodgren *et al.* concluded that the results could not be used to draw firm conclusions on the effectiveness of external inspection.

The Matrix Knowledge group searched the literature in 2010 and found 56 articles that addressed the impact of hospital accreditation [16]. The vast majority of these studies used surveys with standardized questionnaires, and reported staff, patient and stakeholders’ perceptions of impact. Overall they reported a positive impact of accreditation on hospital and professional practice. Only the South African cluster-randomized controlled trial was consistent with the inclusion criteria of our study.

Alkhenizan and Shaw searched the literature in 2009 and included 26 studies that assessed either the general impact of accreditation on hospitals or impact on a single aspect of performance of healthcare services, and on sub-specialty accreditation programs. The authors found a positive effect of accreditation on improving the process of care and clinical outcomes [17]. Sixteen (62 %) of the 26 included studies reported significant positive results attributed to accreditation, mainly related to better compliance with guidelines. Ten studies (38 %) reported weak or no improvement after accreditation. Alkhenizan and Shaw included the one RCT by Salmon *et al.* [18].

### Included primary study

There was one primary study identified that met all of our criteria, the randomized controlled trial from South Africa by Salmon *et al.* [18]. This study was not identified through the database search, but by searching reference lists (snowballing); it was missed by our literature review in 2009. The authors included 20 hospitals in their study. The hospitals were randomly selected and stratified into groups according to hospital size (number of beds). Ten hospitals were randomized to start an accreditation program, while the other 10 served as controls. Two sets of data, before and after measures, were collected by the Council for Health Services Accreditation of Southern Africa (COHSASA), and by independent research teams. Initially, 12 indicators of hospital care quality were identified and used for the first data collection – this number was reduced to eight in the second data collection. Of these indicators, surgical wound infection, time to surgery, neonatal mortality rate and financial solvency were left out due to challenges in data collection. It is unclear whether the four indicators that were abandoned would have influenced the overall magnitude, range of results or conclusions of the study.

The compliance with the COHSASA accreditation standard was found to have increased substantially in the accredited hospitals (*p* < 0.001), whereas the control hospitals maintained their score throughout the study. Eight hospital quality indicators were reported. The nurses’ perceptions of clinical quality was increased in the accredited hospitals (*p* = 0.031); however, the other seven indicators showed little or no effect on the quality indicators; patient satisfaction with care (*p* = 0.484); patient medication education (*p* = 0.395); accessibility of medical records (*p* = 0.492); completeness of medical records (*p* = 0.114); completeness of peri-operative notes (*p* = 0.489); labelling of ward stock (*p* = 0.112); and, composite assessment of hospital sanitation (*p* = 0.641).

### Excluded studies

Sixteen of the 20 studies were excluded after they were independently evaluated by two researchers (GVE, KB). The reasons for exclusion were as follows: four studies had no control groups [21–24]; two performed the study outside hospitals [25, 26]; four studies did not report on the effects of accreditation [27–30]; two studies lacked baseline measurements [31, 32]; one study lacked description of the accreditation intervention [33]; two studies did a comparison of the clinical outcome in accredited hospitals with outcome in non-accredited hospitals, but did not assess the effect of the intervention *per se*. [34, 35]; and, one systematic review conducted a qualitative assessment of healthcare professionals’ attitude toward accreditation, but the effect of the intervention was not assessed [36]. A complete list of the excluded studies and the reasons for their exclusion is presented in Table 4.

**Table 4:** Excluded studies

Reference (country)	Reason cited for exclusion	Aim of study
Al Awa *et al.* 2011 [22], Saudi Arabia	No control group	Determine if patient safety and quality care indicators improve post accreditation
Al Awa *et al.* 2011 [23], Saudi Arabia	No control group	Evaluate nursing perception of care/safety after accreditation
Al Tehewy *et al.* 2009 [26], Egypt	Not in hospital (health units)	Determine the effects of accreditation of non-governmental organizations
Chen *et al.* 2003 [31], USA	Measured outcome at only one point	Identify association between JCAHO accreditation and quality of care for acute myocardial infection
Chuang *et al.* 2009 [27], Australia	Did not measure effect	Propose an integrated research model
du Bois *et al.* 2009 [33], Germany	Review not linked to accreditation	Evaluate the impact of different physician and hospital characteristics on outcome in ovarian cancer patients
Gokenbach *et al.* 2011, USA [24]	No control group	Identify the effects of Magnet accreditation on one hospital
Lichtman *et al.* 2009 [28], USA	Did not measure effects of accreditation	Determine whether hospitals certified had better outcome within the first year of accreditation than non accredited hospitals
Lichtman *et al.* 2011 [32], USA	Measured outcome at only one point	Identify outcomes after ischemic stroke for hospitals with and without TJC certification
Menachemi *et al.* 2008 [25], USA	Not in hospital	Identify quality outcome in accredited and non-accredited ambulatory surgical centers
V Phua *et al.* 2011 [29], Singapore/Asia	Did not evaluate effects of accreditation	Assess compliance to sepsis bundles in intensive care units in Asia
Al-Awa *et al.* 2012 [30], Saudi Arabia	Compares survey results in accredited hospital to international results	Perform an unbiased assessment of the impact of accreditation on patient safety culture
Alkenizan & Shaw 2012 [36], UK,	Review, Qualitative assessment of attitude, did not measure effect	Review the literature of the attitude of healthcare professionals towards accreditation
Bohmer *et al.* 2012 [21], Germany	No controls	Identify to which extent pain management standards was implemented in hospitals after accreditation
Schmaltz *et al.* 2011 [34], USA	Compared the difference in development of accredited vs. non accredited hospitals, not the effects of accreditation	Examine the association between Joint Commission accreditation status and both absolute measures and trends in hospital performance
Nguyen *et al.* 2012 [35], USA	Compared the difference in development of accredited vs. non accredited hospitals, not the effects of accreditation	Analyze the peri-operative outcomes of bariatric surgery performed at accredited vs. non accredited centres

Excluded studies after full text assessment presenting aim of study and reason for exclusion

### Narrative review of excluded studies

We identified one primary study and three systematic reviews. Notably, we had strict inclusion criteria and found few studies that met these strict criteria. A summary of the methods used in the excluded studies is relevant to the discussion on assessing the full measure of complex interventions. Accreditation was addressed in several ways in the publications that failed to fulfil the criteria for inclusion in the present review. Seven of the 16 excluded studies conducted cross-sectional studies comparing patient outcome in accredited and non-accredited hospitals [23, 25, 31, 32, 34, 35]. In general, performance in accredited hospitals was higher than in non-accredited hospitals and showing higher compliance to standards also affecting outcome positively [29]. The study by Lichtman *et al.* identified the risk of selection bias as certified hospitals had better outcome than non-certified hospitals even before the program began [28]. Four studies used survey to assess the staff and patient’ perception of patient safety culture, quality and patient satisfaction pre- and post accreditation [21, 22, 26, 30]. Nurses and patients reported that positive changes in their organization were a result of accreditation, while physicians in general were more sceptical. This is consistent with the findings in Alkhenizan and Shaw’s systematic review studying healthcare professionals’ attitude toward accreditation. Nurses in general were more favourably inclined than physicians indicating the necessity of special education schemes to involve staff in the accreditation process [36]. Another systematic review did not address accreditation directly, but found that physician specialization had effect on outcome of ovarian cancer patients [34]. A study on implementing nurse accreditation in one hospital reports increased staff and patient satisfaction, improved nurse-physician relationship, improved nursing quality and reduced turnover and vacancy rates [24]. The last study aims at proposing an integrated research model of the accreditation and quality measurement/reporting systems, providing more supportive information on the system weakness [27].

## Discussion

In this systematic review, we examined 20 studies involving accreditation and certification aimed at improving patient and organizational outcomes. Because few studies specifically addressed the correlation between accreditation and certification of hospitals and patient outcomes, we could not reach firm conclusions regarding effective strategies in this area.

This is no surprise as accreditation is anticipated as a prototypical example of a complex intervention. Within our classification of interventions, the manner in which the studies carried out specific interventions varied widely. There is complexity in the intervention components as well as in the theoretical background of the intervention, the implementation context, and the targeted outcomes [37]. The literature is dominated by descriptive studies attributing changes in the organization to the accreditation process. The research has ranged from identifying the change in compliance to standards, patient satisfaction, performance indicators, health professionals’ satisfaction and an overall review of the perceptions of accreditation and/or certification among patients, professionals and other stakeholders. Many of the studies we reviewed were heterogeneous, uncontrolled and fraught with confounding variables, adding little clarity or guidance. Despite the lack of convincing evidence there is no reason to believe that accreditation and certification will be abandoned. The lack of documented effect may simply mean that due to the heterogeneity of study design and methods much uncertainty remains regarding its putative effects.

The paucity of evidence is highlighted by our systematic search that revealed variable degrees of rigor. The search identified only one controlled study, the randomized trial from South Africa from 2003. The study, however, is weak scientifically, and does not address morbidity or patient safety measures well enough to support any conclusions across a wide range of safety systems examined.

The methodological challenges of measuring the effects of accreditation/certification are increased by the complexity of the hospital organizations and their heterogeneous components. It is unclear what elements are being subjected to assessment [38, 39]. The UK Medical Research Council points out that it is hard to identify the “active ingredient” of complex interventions such as falls prevention or hand washing campaigns, as these interventions comprise many separate, multi-level and concurrent elements [40]. Furthermore, the interventions are interpreted in many ways and are used in different settings which strongly complicate the evaluation of the effects [41, 42]. Lessons can be learned from non-controlled studies such as cross-sectional studies. Comparison between accredited and non-accredited hospitals yields important information about potential differences between these hospitals, but cannot provide information about the observed variations, and whether the results are transferable to other settings.

It is noteworthy that there was a low level of methodological rigor in most of the studies included in this review, as outcome measures were ambiguous and only limited operational details were reported. Significant methodological challenges such as self-selection and lack of robust controls undermine the ability to extrapolate or infer from the published literature if these effects were caused by accreditation and/or certification [43]. Even though our systematic review was conducted carefully adhering to the Cochrane guidelines, we were unable to find conclusive evidence on accreditation and certification. Some studies surveyed staff, stakeholders or other hospital representatives before, during and after a certification and/or accreditation process. Some studies show higher quality in accredited hospitals when compared to non-accredited hospitals, but it is uncertain if this is the result of accreditation, self-selection or is due to other extraneous factors.

Working with predetermined inclusion criteria allowed a specialized literature search which generally increases the chances of finding all relevant studies although it only identifies the published literature. Reports of studies that are only posted on the web pages of organizations or stakeholders (grey literature) are more difficult to find. Notably, the randomized controlled trial from South Africa was only available as grey literature and was not identified through a systematic literature search of electronic databases. Although unlikely, it is possible that there may be other studies that the present or other reviews have missed.

Our study has several limitations. An unavoidable limitation of systematic reviews is that they may appear out-dated rapidly as new studies are published; hence, our review only included recently published systematic reviews. Notably, we repeated the search in July 2014 to ensure that we captured any new studies. Future investigations might control for case mix and time trends, employ suitable comparison groups, and consider other analytic approaches for analyzing time series data such as interrupted time series data, or ARIMA methods [44]. Interrupted time series analyses, Bayesian analysis and ARIMA may be suited for adjusting for clustering of effects within sites, while accounting for patient-level effects, and site-level structural measures. Studies addressing how and why the interventions might work, rather than just the effects of the intervention, might provide valuable information on complex interventions [39].

## Conclusions

Hospitals are now faced with the challenge of improving their patient outcomes and reliability. Our study provides a comprehensive overview of the effects of accreditation and/or certification of hospitals on quality and patient safety outcomes and concludes that due to scant evidence, no conclusions could be reached to support its effectiveness. The accreditation programs require substantial financial and labor investments, and distract healthcare teams from their primary clinical goals. Accordingly further research on the clinical impact of these programs is needed, and it is important to weigh the transactional opportunity and financial costs of accreditation against other financial investments in quality improvement interventions [45–48]. Furthermore, we found little guidance demonstrating the cost effectiveness of accreditation and/or certification.

In summary, we found that the proven role of accreditation and certification in improving patient and organizational outcomes remain largely undefined. Accreditation and certification is a thriving industry and there are many interested stakeholders who may profit on promoting these services despite the lack of robust evidence of their effectiveness [49, 50]. Finally, because hospitals are expending resources on accreditation and/or certification they may not be able to address other, more pressing patient safety issues [51]. There is little reason to believe however that accreditation or certification will be abandoned because of the lack of empirical evidence of its effects, so future contributions should probably focus on what aspects of accreditation serve a useful purpose, rather than focusing on “does it work”.

Before planning further studies to evaluate impact of accreditation and certification efforts, a more thorough and nuanced analysis of the available evidence about which components of accreditation/certification seem to be most effective in enabling patient centered, high quality and safer outcomes should be performed [37].

## Additional files

Additional file 1:
**Complete search strategy January 2013.** Complete search strategy performed in PubMed (from 1948), EMBASE (from 1980), CRD, and the Cochrane Library, including the Cochrane Database of Systematic Reviews (CDSR), Database of Abstracts of Reviews of Effects (DARE) and Health Technology Assessment Database (HTA) January 2013. Additional file 2:
**Complete search strategy July 2014.** Complete search strategy performed in PubMed (from 1948), EMBASE (from 1980), CRD, and the Cochrane Library, including the Cochrane Database of Systematic Reviews (CDSR), Database of Abstracts of Reviews of Effects (DARE) and Health Technology Assessment Database (HTA) July 2014. Additional file 3:
**PRISMA 2009 Checklist. PRISMA 2009 Checklist.** Transparent reporting of systematic reviews and meta-analyses. Prisma-statement.org.
