# Supplementary material for: A systematic review of hospital accreditation: the challenges of measuring complex intervention effects
Source: BMC Health Serv Res. 2015 Jul 23;15:280. doi: 10.1186/s12913-015-0933-x (PMC4511980; doi:10.1186/s12913-015-0933-x)
Supplement: Additional file 1: — Complete search strategy January 2013. Complete search strategy performed in PubMed (from 1948), EMBASE (from 1980), CRD, and the Cochrane Library, including the Cochrane Database of Systematic Reviews (CDSR), Database of Abstracts of Reviews of Effects (DARE) and Health Technology Assessment Database (HTA) January 2013. [file 12913_2015_933_MOESM1_ESM.doc]

Additional file 1. Search Strategy

### January 18, 2013: search strategy in the Cochrane Library

**Database:** The Cochrane Library:

Cochrane Database of Systematic Reviews, Issue 12 of 12, December 2012

DARE, Cochrane Central Register of Controlled Trials of Technology Assessments, Issue 4 of 4 October 2012

#1 MeSH descriptor Hospitals explode all trees 2423

#2 MeSH descriptor Hospital Departments explode all trees 2516

#3 MeSH descriptor Hospital Units explode all trees 2562

#4 MeSH descriptor Rehabilitation Centers, this term only 203

#5 (hospital or hospitals or ward or wards or (medical next clinic*) or (private next clinic*) or ((academic or university) next medical next (center* or centre*)) or (university next health next facilit*) or (health next facilit* next (department* or unit*)) or (cancer next care next (facilit* or unit*)) or (cancer near/2 (center* or centre*)) or (cancer next (clinic* or institute*)) or (oncology next service*) or ((oncology or oncologic) next care next unit*) or (cardiac next care next (facilit* or unit*)) or (cardiology next service*) or ((coronary or stroke) near/2 unit*) or ccu or ccus or ((cardiologic or cva) next unit*) or (heart next (center* or centre*))):ti,ab,kw 45147

#6 (hospice* or (pain next (clinic* or center* or centre*)) or (acute next pain next service*) or (pain next relief next unit*) or (rehabilitation next (center* or centre* or clinic* or department* or service* or unit*)) or (rehab next (center* or centre*)) or (psychiatric next (clinic* or department* or unit* or (health next facilit*))) or (mental next (institution* or (health next facilit*))) or (psychiatry next unit*) or (day next clinic*) or surgicenter* or surgicentre* or ((surgery or surgical) next (center* or centre* or department*)) or (surgical next service*) or ((ambulatory or outpatient) next (surgery or surgical) next facilit*) or (geriatric next (center* or centre* or clinic* or institute*))):ti,ab,kw 2543

#7 (child near/2 clinic*) or (children next institution*) or (child next health next (center* or centre*)) or ((pediatric or paediatric) near/2 (center* or centre*)) or ((pediatric or paediatric) next (clinic* or unit*)) or ((delivery or delivering or labo*r) next room*) or (delivery next unit*) or (maternity next (clinic* or home* or unit*)) or (midwifery next service*) or ((birth or birthing) next (center* or centre*)) or ((gynecology or obstetrics) next department*) or (obstetric* next service*) or ((operation or operating) next room*) or ((operation or operating or surgical) next (theater* or theatre*)):ti,ab,kw 4407

#8 (outpatient next (department* or clinic* or unit* or service*)) or (ambulatory next care next facilit*) or (outdoor next clinic*) or pol*clinic or pol*clinics or ((radiology or x-ray or radiodiagnosis or radiography or radiological or radiotherapy or roentgen) next department*) or (roentgen next facilit*) or (radiology next service*) or (recovery next room*) or (((post next anesthesia) or postanesthesia) next care next unit*) or pacu or pacus or ((emergency or "a & e" or "a&e" or "a and e" or casualty) next department*) or (("a & e" or "a&e" or "a and e") next service*) or (emergency next (room* or unit*)):ti,ab,kw 8149

#9 (trauma next (center* or centre* or unit*)) or (intensive next (care or therapy) next unit*) or icu or icus or itu or itus or picu or picus or pitu or pitus or nicu or nicus or nitu or nitus or itun or ituns or ((burn or burns) next (unit* or center* or centre*)) or (admitting next department*) or (medical next (record or records) next (department* or service*)) or (health next information next management next service*) or (nuclear next medicine next department*) or ((hemodialysis or (renal next dialysis)) next unit*) or ((self or minimal or cooperative) next care next unit*) or (observation next unit*) or (pre next (admission or admitting) next unit*) or (step next down next unit*):ti,ab,kw 7192

#10 (medical next assessment next unit*) or (anesthesia near/2 department*) or (anesthesiology next service*) or (occupational next therap* next (department* or service*)) or (pathology next department*) or (physical next therap* next (department* or service*)) or (respiratory next therap* next (department* or service*)) or (respiratory next care next unit*) or (social next work next department*) or (urology next department*) or (venereal next disease next department*) or (endoscopy next department*) or ((clinical or nuclear) next pharmacy next service*) or (inpatient next pharmac*) or (((intravenous next therap*) or (iv next therapy)) next department*) or (nursing next unit*):ti,ab,kw 285

#11 (#1 OR #2 OR #3 OR #4 OR #5 OR #6 OR #7 OR #8 OR #9 OR #10) 57741

#12 MeSH descriptor Accreditation explode all trees 11

#13 MeSH descriptor Certification, this term only 35

#14 (accr* or "jcaho" or "jcia" or "urac" or "equip" or "carf" or "evaluation and quality improvement program" or ((international next organi*ation) near/2 standard*) or (international next standard* next organi*ation) or (iso near/10 (certif* or "9001" or standard* or system* or qualified or quality or based or assessment*))):ti,ab,kw 3413

#15 “Joint Commission on Accreditation of Healthcare Organizations":ti,ab,kw 5

#16 (#12 OR #13 OR #14 OR #15) 3446

#17 (#11 AND #16) 73

#18 (#17), from 2011 to 2013, in Cochrane Reviews (Reviews and Protocols), Other Reviews, Trials and Technology Assessments 65

### January 18, 2013: search strategy in Ovid Embase

**Database:** Embase 1980 to 2013 Week 02,

**Search terms**

1. cancer center/ or hospice/ or pain clinic/ or rehabilitation center/ or exp hospital/

2. hospital$1.tw.

3. ward$1.tw.

4. medical clinic$.tw.

5. private clinic$.tw.

6. ((academic or university) adj medical adj (center$ or centre$)).tw.

7. university health facilit$.tw.

8. (health facilit$ adj (department$ or unit$)).tw.

9. (cancer care adj (facilit$ or unit$)).tw.

10. (cancer adj2 (center$ or centre$)).tw.

11. (cancer adj (clinic$ or institute$)).tw.

12. oncology service$.tw.

13. ((oncology or oncologic) adj care unit$).tw.

14. (cardiac care adj (facilit$ or unit$)).tw.

15. cardiology service$.tw.

16. ((coronary or stroke) adj2 unit$).tw.

17. ccu$1.tw.

18. ((cardiologic or cva) adj unit$).tw.

19. (heart adj (center$ or centre$)).tw.

20. hospice$.tw.

21. (pain adj (clinic$ or center$ or centre$)).tw.

22. acute pain service$.tw.

23. pain relief unit$.tw.

24. (rehabilitation adj (center$ or centre$ or clinic$ or department$ or service$ or unit$)).tw.

25. (rehab adj (center$ or centre$)).tw.

26. (psychiatric adj (clinic$ or department$ or unit$ or health facilit$)).tw.

27. (mental adj (institution$ or health facilit$)).tw.

28. psychiatry unit$.tw.

29. day clinic$.tw.

30. (surgicenter$ or surgicentre$).tw.

31. ((surgery or surgical) adj (center$ or centre$ or department$)).tw.

32. surgical service$.tw.

33. ((ambulatory or outpatient) adj (surgery or surgical) adj facilit$).tw.

34. (geriatric adj (center$ or centre$ or clinic$ or institute$)).tw.

35. (child adj2 clinic$).tw.

36. children institution$.tw.

37. (child health adj (center$ or centre$)).tw.

38. ((pediatric or paediatric) adj2 (center$ or centre$)).tw.

39. ((pediatric or paediatric) adj (clinic$ or unit$)).tw.

40. ((delivery or delivering or labo?r) adj room$).tw.

41. delivery unit$.tw.

42. (maternity adj (clinic$ or home$ or unit$)).tw.

43. midwifery service$.tw.

44. ((birth or birthing) adj (center$ or centre$)).tw.

45. ((gynecology or obstetrics) adj department$).tw.

46. (obstetric$ adj service$).tw.

47. ((operation or operating) adj room$).tw.

48. ((operation or operating or surgical) adj (theater$ or theatre$)).tw.

49. (outpatient adj (department$ or clinic$ or unit$ or service$)).tw.

50. ambulatory care facilit$.tw.

51. outdoor clinic$.tw.

52. pol#clinic$1.tw.

53. ((radiology or x-ray or radiodiagnosis or radiography or radiological or radiotherapy or roentgen) adj department$).tw.

54. roentgen facilit$.tw.

55. radiology service$.tw.

56. recovery room$.tw.

57. ((post anesthesia or postanesthesia) adj care unit$).tw.

58. pacu$1.tw.

59. ((emergency or "a & e" or "a&e" or "a and e" or casualty) adj (department$ or ward$)).tw.

60. (("a & e" or "a&e" or "a and e") adj service$).tw.

61. (emergency adj (room$ or unit$)).tw.

62. (trauma adj (center$ or centre$ or unit$)).tw.

63. (intensive adj (care or therapy) adj unit$).tw.

64. (icu$1 or itu$1 or picu$1 or pitu$1 or nicu$1 or nitu$1 or itun$1).tw.

65. (burn$1 adj (unit$ or center$ or centre$)).tw.

66. admitting department$.tw.

67. (medical record$1 adj (department$ or service$)).tw.

68. health information management service$.tw.

69. nuclear medicine department$.tw.

70. ((hemodialysis or renal dialysis) adj unit$).tw.

71. ((self or minimal or cooperative) adj care unit$).tw.

72. observation unit$.tw.

73. (pre adj (admission or admitting) adj unit$).tw.

74. step down unit$.tw.

75. medical assessment unit$.tw.

76. (anesthesia adj2 department$).tw.

77. anesthesiology service$.tw.

78. (occupational therap$ adj (department$ or service$)).tw.

79. pathology department$.tw.

80. (physical therap$ adj (department$ or service$)).tw.

81. (respiratory therap$ adj (department$ or service$)).tw.

82. respiratory care unit$.tw.

83. social work department$.tw.

84. urology department$.tw.

85. venereal disease department$.tw.

86. endoscopy department$.tw.

87. ((clinical or nuclear) adj pharmacy service$).tw.

88. inpatient pharmac$.tw.

89. ((intravenous therap$ or iv therapy) adj department$).tw.

90. nursing unit$.tw.

91. or/1-90

92. accreditation/ or certification/

93. accredit$.tw.

94. (jcaho or jcia or urac or equip or carf).tw.

95. "Joint Commission on Accreditation of Healthcare Organizations".tw.

96. "evaluation and quality improvement program".tw.

97. (international organi#ation adj2 standard$).tw.

98. (international standard$ adj organi#ation).tw.

99. (iso adj10 (certif$ or "9001" or standard$ or system$ or qualified or quality or based or assessment$)).tw.

100. or/92-99

101. 91 and 100

102. limit 101 to "reviews (best balance of sensitivity and specificity)"

103. 2011$.em,dp,dd,yr.

104. 2012$.em,dp,dd,yr.

105. 2013$.em,dp,dd,yr.

106. or/103-105

107. 102 and 106

108. clinical trial/

109. randomized controlled trial/

110. randomization/

111. double blind procedure/

112. single blind procedure/

113. crossover procedure/

114. placebo/

115. placebo$.tw.

116. randomi?ed controlled trial$.tw.

117. rct.tw.

118. random allocation.tw.

119. randomly allocated.tw.

120. allocated randomly.tw.

121. (allocated adj2 random).tw.

122. single blind$.tw.

123. double blind$.tw.

124. ((treble or triple) adj blind$).tw.

125. prospective study/

126. or/108-125

127. case study/

128. case report.tw.

129. abstract report/

130. letter/

131. human/

132. nonhuman/

133. animal/

134. animal experiment/

135. 132 or 133 or 134

136. 135 not (131 and 135)

137. or/127-130,136

138. 126 not 137

139. 101 and 138

140. 106 and 139

141. evaluation.sh.

142. evaluation stud$.tw.

143. "types of study".sh.

144. intervention study.sh.

145. (intervention$ adj (stud$ or trial$)).tw.

146. comparative study.sh.

147. comparative stud$.tw.

148. experimental study.sh.

149. experimental stud$.tw.

150. (time adj series).tw.

151. (pre test or pretest or post test or posttest).tw.

152. or/141-151

153. 101 and 152

154. 106 and 153

155. 107 or 140 or 154

**Database:** Ovid MEDLINE(R) 1948 to Present

**Dato**: 18.1.2013.

**Search terms**

1. exp hospital departments/ or exp hospital units/ or exp hospitals/ or rehabilitation centers/

2. hospital$1.tw.

3. ward$1.tw.

4. medical clinic$.tw.

5. private clinic$.tw.

6. ((academic or university) adj medical adj (center$ or centre$)).tw.

7. university health facilit$.tw.

8. (health facilit$ adj (department$ or unit$)).tw.

9. (cancer care adj (facilit$ or unit$)).tw.

10. (cancer adj2 (center$ or centre$)).tw.

11. (cancer adj (clinic$ or institute$)).tw.

12. oncology service$.tw.

13. ((oncology or oncologic) adj care unit$).tw.

14. (cardiac care adj (facilit$ or unit$)).tw.

15. cardiology service$.tw.

16. ((coronary or stroke) adj2 unit$).tw.

17. ccu$1.tw.

18. ((cardiologic or cva) adj unit$).tw.

19. (heart adj (center$ or centre$)).tw.

20. hospice$.tw.

21. (pain adj (clinic$ or center$ or centre$)).tw.

22. acute pain service$.tw.

23. pain relief unit$.tw.

24. (rehabilitation adj (center$ or centre$ or clinic$ or department$ or service$ or unit$)).tw.

25. (rehab adj (center$ or centre$)).tw.

26. (psychiatric adj (clinic$ or department$ or unit$ or health facilit$)).tw.

27. (mental adj (institution$ or health facilit$)).tw.

28. psychiatry unit$.tw.

29. day clinic$.tw.

30. (surgicenter$ or surgicentre$).tw.

31. ((surgery or surgical) adj (center$ or centre$ or department$)).tw.

32. surgical service$.tw.

33. ((ambulatory or outpatient) adj (surgery or surgical) adj facilit$).tw.

34. (geriatric adj (center$ or centre$ or clinic$ or institute$)).tw.

35. (child adj2 clinic$).tw.

36. children institution$.tw.

37. (child health adj (center$ or centre$)).tw.

38. ((pediatric or paediatric) adj2 (center$ or centre$)).tw.

39. ((pediatric or paediatric) adj (clinic$ or unit$)).tw.

40. ((delivery or delivering or labo?r) adj room$).tw.

41. delivery unit$.tw.

42. (maternity adj (clinic$ or home$ or unit$)).tw.

43. midwifery service$.tw.

44. ((birth or birthing) adj (center$ or centre$)).tw.

45. ((gynecology or obstetrics) adj department$).tw.

46. (obstetric$ adj service$).tw.

47. ((operation or operating) adj room$).tw.

48. ((operation or operating or surgical) adj (theater$ or theatre$)).tw.

49. (outpatient adj (department$ or clinic$ or unit$ or service$)).tw.

50. ambulatory care facilit$.tw.

51. outdoor clinic$.tw.

52. pol#clinic$1.tw.

53. ((radiology or x-ray or radiodiagnosis or radiography or radiological or radiotherapy or roentgen) adj department$).tw.

54. roentgen facilit$.tw.

55. radiology service$.tw.

56. recovery room$.tw.

57. ((post anesthesia or postanesthesia) adj care unit$).tw.

58. pacu$1.tw.

59. ((emergency or "a & e" or "a&e" or "a and e" or casualty) adj department$).tw.

60. (("a & e" or "a&e" or "a and e") adj service$).tw.

61. (emergency adj (room$ or unit$)).tw.

62. (trauma adj (center$ or centre$ or unit$)).tw.

63. (intensive adj (care or therapy) adj unit$).tw.

64. (icu$1 or itu$1 or picu$1 or pitu$1 or nicu$1 or nitu$1 or itun$1).tw.

65. (burn$1 adj (unit$ or center$ or centre$)).tw.

66. admitting department$.tw.

67. (medical record$1 adj (department$ or service$)).tw.

68. health information management service$.tw.

69. nuclear medicine department$.tw.

70. ((hemodialysis or renal dialysis) adj unit$).tw.

71. ((self or minimal or cooperative) adj care unit$).tw.

72. observation unit$.tw.

73. (pre adj (admission or admitting) adj unit$).tw.

74. step down unit$.tw.

75. medical assessment unit$.tw.

76. (anesthesia adj2 department$).tw.

77. anesthesiology service$.tw.

78. (occupational therap$ adj (department$ or service$)).tw.

79. pathology department$.tw.

80. (physical therap$ adj (department$ or service$)).tw.

81. (respiratory therap$ adj (department$ or service$)).tw.

82. respiratory care unit$.tw.

83. social work department$.tw.

84. urology department$.tw.

85. venereal disease department$.tw.

86. endoscopy department$.tw.

87. ((clinical or nuclear) adj pharmacy service$).tw.

88. inpatient pharmac$.tw.

89. ((intravenous therap$ or iv therapy) adj department$).tw.

90. nursing unit$.tw.

91. or/1-90

92. exp accreditation/ or certification/

93. accredit$.tw.

94. (jcaho or jcia or urac or equip or carf).tw.

95. "Joint Commission on Accreditation of Healthcare Organizations".tw.

96. "evaluation and quality improvement program".tw.

97. (international organi#ation adj2 standard$).tw.

98. (international standard$ adj organi#ation).tw.

99. (iso adj10 (certif$ or "9001" or standard$ or system$ or qualified or quality or based or assessment$)).tw.

100. or/92-99

101. 91 and 100

102. limit 101 to "reviews (best balance of sensitivity and specificity)”

103. 2011$.ed,ep,yr,dp.

104. 2012$.ed,ep,yr,dp.

105. 2013$.ed,ep,yr,dp.

106. or/103-105

107. 102 and 106

108. randomized controlled trial.pt.

109. controlled clinical trial.pt.

110. randomi#ed.ab,ti.

111. placebo.ab,ti.

112. clinical trials as topic.sh.

113. randomly.ab,ti.

114. trial.ti,ab.

115. or/108-114

116. exp animals/ not humans.sh.

117. 115 not 116

118. 101 and 117

119. 106 and 118

120. evaluation studies.pt,sh.

121. evaluation stud$.tw.

122. intervention studies.sh.

123. intervention stud$.tw.

124. comparative study.pt,sh.

125. comparative stud$.tw.

126. experimental stud$.tw.

127. (time adj series).tw.

128. (pre test or pretest or post test or posttest).tw.

129. or/120-128

130. 101 and 129

131. 106 and 130

132. 107 or 119 or 131
