# Supplementary material for: A systematic review of hospital accreditation: the challenges of measuring complex intervention effects
Source: BMC Health Serv Res. 2015 Jul 23;15:280. doi: 10.1186/s12913-015-0933-x (PMC4511980; doi:10.1186/s12913-015-0933-x)
Supplement: Additional file 2: — Complete search strategy July 2014. Complete search strategy performed in PubMed (from 1948), EMBASE (from 1980), CRD, and the Cochrane Library, including the Cochrane Database of Systematic Reviews (CDSR), Database of Abstracts of Reviews of Effects (DARE) and Health Technology Assessment Database (HTA) July 2014. [file 12913_2015_933_MOESM2_ESM.docx]

**Database: Central, HTA, DARE (via Cochrane Library)**

**Search date: July 1, 2014**

| #1 | MeSH descriptor: [Hospitals] explode all trees | 2958 |
| --- | --- | --- |
| #2 | MeSH descriptor: [Hospital Departments] explode all trees | 2988 |
| #3 | MeSH descriptor: [Hospital Units] explode all trees | 3107 |
| #4 | MeSH descriptor: [Rehabilitation Centers] this term only | 243 |
| #5 | (hospital or hospitals or ward or wards or (medical next clinic*) or (private next clinic*) or ((academic or university) next medical next (center* or centre*)) or (university next health next facilit*) or (health next facilit* next (department* or unit*)) or (cancer next care next (facilit* or unit*)) or (cancer near/2 (center* or centre*)) or (cancer next (clinic* or institute*)) or (oncology next service*) or ((oncology or oncologic) next care next unit*) or (cardiac next care next (facilit* or unit*)) or (cardiology next service*) or ((coronary or stroke) near/2 unit*) or ccu or ccus or ((cardiologic or cva) next unit*) or (heart next (center* or centre*))) (Word variations have been searched) | 176692 |
| #6 | (hospice* or (pain next (clinic* or center* or centre*)) or (acute next pain next service*) or (pain next relief next unit*) or (rehabilitation next (center* or centre* or clinic* or department* or service* or unit*)) or (rehab next (center* or centre*)) or (psychiatric next (clinic* or department* or unit* or (health next facilit*))) or (mental next (institution* or (health next facilit*))) or (psychiatry next unit*) or (day next clinic*) or surgicenter* or surgicentre* or ((surgery or surgical) next (center* or centre* or department*)) or (surgical next service*) or ((ambulatory or outpatient) next (surgery or surgical) next facilit*) or (geriatric next (center* or centre* or clinic* or institute*))) (Word variations have been searched) | 6758 |
| #7 | (child near/2 clinic*) or (children next institution*) or (child next health next (center* or centre*)) or ((pediatric or paediatric) near/2 (center* or centre*)) or ((pediatric or paediatric) next (clinic* or unit*)) or ((delivery or delivering or labo*r) next room*) or (delivery next unit*) or (maternity next (clinic* or home* or unit*)) or (midwifery next service*) or ((birth or birthing) next (center* or centre*)) or ((gynecology or obstetrics) next department*) or (obstetric* next service*) or ((operation or operating) next room*) or ((operation or operating or surgical) next (theater* or theatre*)) (Word variations have been searched) | 9139 |
| #8 | (outpatient next (department* or clinic* or unit* or service*)) or (ambulatory next care next facilit*) or (outdoor next clinic*) or pol*clinic or pol*clinics or ((radiology or x-ray or radiodiagnosis or radiography or radiological or radiotherapy or roentgen) next department*) or (roentgen next facilit*) or (radiology next service*) or (recovery next room*) or (((post next anesthesia) or postanesthesia) next care next unit*) or pacu or pacus or ((emergency or "a & e" or "a&e" or "a and e" or casualty) next department*) or (("a & e" or "a&e" or "a and e") next service*) or (emergency next (room* or unit*)) (Word variations have been searched) | 13087 |
| #9 | (trauma next (center* or centre* or unit*)) or (intensive next (care or therapy) next unit*) or icu or icus or itu or itus or picu or picus or pitu or pitus or nicu or nicus or nitu or nitus or itun or ituns or ((burn or burns) next (unit* or center* or centre*)) or (admitting next department*) or (medical next (record or records) next (department* or service*)) or (health next information next management next service*) or (nuclear next medicine next department*) or ((hemodialysis or (renal next dialysis)) next unit*) or ((self or minimal or cooperative) next care next unit*) or (observation next unit*) or (pre next (admission or admitting) next unit*) or (step next down next unit*) (Word variations have been searched) | 11837 |
| #10 | (medical next assessment next unit*) or (anesthesia near/2 department*) or (anesthesiology next service*) or (occupational next therap* next (department* or service*)) or (pathology next department*) or (physical next therap* next (department* or service*)) or (respiratory next therap* next (department* or service*)) or (respiratory next care next unit*) or (social next work next department*) or (urology next department*) or (venereal next disease next department*) or (endoscopy next department*) or ((clinical or nuclear) next pharmacy next service*) or (inpatient next pharmac*) or (((intravenous next therap*) or (iv next therapy)) next department*) or (nursing next unit*) (Word variations have been searched) | 7454 |
| #11 | (#1 or #2 or #3 or #4 or #5 or #6 or #7 or #8 or #9 or #10) | 195107 |
| #12 | MeSH descriptor: [Accreditation] explode all trees | 21 |
| #13 | MeSH descriptor: [Certification] this term only | 37 |
| #14 | (accr* or "jcaho" or "jcia" or "urac" or "equip" or "carf" or "evaluation and quality improvement program" or ((international next organi*ation) near/2 standard*) or (international next standard* next organi*ation) or (iso near/10 (certif* or "9001" or standard* or system* or qualified or quality or based or assessment*))) (Word variations have been searched) | 5250 |
| #15 | "Joint Commission on Accreditation of Healthcare Organizations" (Word variations have been searched) | 12 |
| #16 | (#12 or #13 or #14 or #15) | 5284 |
| #17 | (#11 and #16) Publication Year from 2013 to 2014, in Other Reviews, Trials and Technology Assessments | 78 |

**Database: Centre for Reviews and Dissemination**

**Search date: July 1, 2014**

| 1 | MeSH DESCRIPTOR Hospital Departments EXPLODE ALL TREES | 694 |
| --- | --- | --- |
| 2 | MeSH DESCRIPTOR Hospital Units EXPLODE ALL TREES | 634 |
| 3 | MeSH DESCRIPTOR Hospitals EXPLODE ALL TREES | 693 |
| 4 | MeSH DESCRIPTOR Rehabilitation Centers EXPLODE ALL TREES | 91 |
| 5 | #1 OR #2 OR #3 OR #4 | 1921 |
| 6 | ((hospital* or ward* or clinic* or medical cent* or health facilit* or care facilit* care unit* or cancer cent* or cancer institute* or oncology service* or cardiology service* or coronary unit* or stroke unit* or ccu* or cardiologic unit* or cva unit* or heart cent*)) | 54344 |
| 7 | ((hospice* or pain cent* or pain service* or pain unit* or rehab* cent* or rehab* department* or rehab* service* or rehab* unit* or psychiatric department* or psychiatric unit* or psychiatric facilit* or mental institution* or mental facilit* or psychiatry unit* or surgicent* or surg* cent* or surg* department* or surg* service* or surg* facilit* or geriatric cent* or geriatric institute*)) | 473 |
| 8 | ((children institution* or health cent* or pediatric cent* or paediatric cent* or pediatric unit* or paediatric unit* or deliver* room* or deliver* unit* or labour room* or labor room* or maternity home* or maternity unit* or midwifery service* or birth* cent* or gynecology department* or obstetrics department* or obstetric* service* or operati* room* or operati* theat* or surgical theat*)) | 954 |
| 9 | ((outpatient department* or outpatient unit* or outpatient service* or policlinic* or polyclinic* or radiolog* department* or x-ray department* or radiodiagnosis department* or radiography department* or radiotherapy department* or roentgen department* or roentgen facilit* or radiology service* or recovery room* or pacu* emergency department* or a & e department* or a&e department* a and e department* or casualty department* or a & e service* or a&e service* or a and e service* or emergency room* or emergency unit*)) | 16 |
| 10 | ((trauma cent* or trauma unit* or therapy unit* or icu* or itu* or picu* or pitu* or nicu* or nitu* or itun* or burn* unit* or burn* cent* or admitting department* or record* department* or record* service* or management service* or medicine department* or hemodialysis unit* or dialysis unit* or observation unit* or admission unit* or admitting unit* or step down unit*)) | 976 |
| 11 | ((medical assessment unit* or anesthesia department* or anesthesiology service* or therap* department* or therap* service* or pathology department* or social work department* or urology department* or disease department* or endoscopy department* or pharmacy service* or inpatient pharmac* or nursing unit*)) | 208 |
| 12 | #6 OR #7 OR #8 OR #9 OR #10 OR #11 | 54483 |
| 13 | MeSH DESCRIPTOR Accreditation EXPLODE ALL TREES | 8 |
| 14 | MeSH DESCRIPTOR Certification EXPLODE ALL TREES | 3 |
| 5 | #13 OR #14 | 10 |
| 16 | ((accredit* or jcaho or jcia or urac or equip or carf or quality improvement program* or international organi* or international standard* or iso)) | 408 |
| 17 | ((joint commission on accreditation of healthcare organizations)) | 0 |
| 18 | #15 OR #16 OR #17 | 409 |
| 19 | #12 AND #18 | 278 |
| 20 | * IN DARE, HTA FROM 2013 TO 2014 | 11702 |
| 21 | #19 AND #20 | 18 |

**Database: Cochrane Database of Systematic Reviews**

**Search date: July 1, 2014**

| #1 | MeSH descriptor: [Hospitals] explode all trees | 2958 |
| --- | --- | --- |
| #2 | MeSH descriptor: [Hospital Departments] explode all trees | 2988 |
| #3 | MeSH descriptor: [Hospital Units] explode all trees | 3107 |
| #4 | MeSH descriptor: [Rehabilitation Centers] this term only | 243 |
| #5 | (hospital or hospitals or ward or wards or (medical next clinic*) or (private next clinic*) or ((academic or university) next medical next (center* or centre*)) or (university next health next facilit*) or (health next facilit* next (department* or unit*)) or (cancer next care next (facilit* or unit*)) or (cancer near/2 (center* or centre*)) or (cancer next (clinic* or institute*)) or (oncology next service*) or ((oncology or oncologic) next care next unit*) or (cardiac next care next (facilit* or unit*)) or (cardiology next service*) or ((coronary or stroke) near/2 unit*) or ccu or ccus or ((cardiologic or cva) next unit*) or (heart next (center* or centre*))):ti,ab,kw (Word variations have been searched) | 55339 |
| #6 | (hospice* or (pain next (clinic* or center* or centre*)) or (acute next pain next service*) or (pain next relief next unit*) or (rehabilitation next (center* or centre* or clinic* or department* or service* or unit*)) or (rehab next (center* or centre*)) or (psychiatric next (clinic* or department* or unit* or (health next facilit*))) or (mental next (institution* or (health next facilit*))) or (psychiatry next unit*) or (day next clinic*) or surgicenter* or surgicentre* or ((surgery or surgical) next (center* or centre* or department*)) or (surgical next service*) or ((ambulatory or outpatient) next (surgery or surgical) next facilit*) or (geriatric next (center* or centre* or clinic* or institute*))):ti,ab,kw (Word variations have been searched) | 3179 |
| #7 | (child near/2 clinic*) or (children next institution*) or (child next health next (center* or centre*)) or ((pediatric or paediatric) near/2 (center* or centre*)) or ((pediatric or paediatric) next (clinic* or unit*)) or ((delivery or delivering or labo*r) next room*) or (delivery next unit*) or (maternity next (clinic* or home* or unit*)) or (midwifery next service*) or ((birth or birthing) next (center* or centre*)) or ((gynecology or obstetrics) next department*) or (obstetric* next service*) or ((operation or operating) next room*) or ((operation or operating or surgical) next (theater* or theatre*)):ti,ab,kw (Word variations have been searched) | 6823 |
| #8 | (outpatient next (department* or clinic* or unit* or service*)) or (ambulatory next care next facilit*) or (outdoor next clinic*) or pol*clinic or pol*clinics or ((radiology or x-ray or radiodiagnosis or radiography or radiological or radiotherapy or roentgen) next department*) or (roentgen next facilit*) or (radiology next service*) or (recovery next room*) or (((post next anesthesia) or postanesthesia) next care next unit*) or pacu or pacus or ((emergency or "a & e" or "a&e" or "a and e" or casualty) next department*) or (("a & e" or "a&e" or "a and e") next service*) or (emergency next (room* or unit*)):ti,ab,kw (Word variations have been searched) | 9819 |
| #9 | (trauma next (center* or centre* or unit*)) or (intensive next (care or therapy) next unit*) or icu or icus or itu or itus or picu or picus or pitu or pitus or nicu or nicus or nitu or nitus or itun or ituns or ((burn or burns) next (unit* or center* or centre*)) or (admitting next department*) or (medical next (record or records) next (department* or service*)) or (health next information next management next service*) or (nuclear next medicine next department*) or ((hemodialysis or (renal next dialysis)) next unit*) or ((self or minimal or cooperative) next care next unit*) or (observation next unit*) or (pre next (admission or admitting) next unit*) or (step next down next unit*):ti,ab,kw (Word variations have been searched) | 9117 |
| #10 | (medical next assessment next unit*) or (anesthesia near/2 department*) or (anesthesiology next service*) or (occupational next therap* next (department* or service*)) or (pathology next department*) or (physical next therap* next (department* or service*)) or (respiratory next therap* next (department* or service*)) or (respiratory next care next unit*) or (social next work next department*) or (urology next department*) or (venereal next disease next department*) or (endoscopy next department*) or ((clinical or nuclear) next pharmacy next service*) or (inpatient next pharmac*) or (((intravenous next therap*) or (iv next therapy)) next department*) or (nursing next unit*):ti,ab,kw (Word variations have been searched) | 337 |
| #11 | (#1 or #2 or #3 or #4 or #5 or #6 or #7 or #8 or #9 or #10) | 72070 |
| #12 | MeSH descriptor: [Accreditation] explode all trees | 21 |
| #13 | MeSH descriptor: [Certification] this term only | 37 |
| #14 | (accr* or "jcaho" or "jcia" or "urac" or "equip" or "carf" or "evaluation and quality improvement program" or ((international next organi*ation) near/2 standard*) or (international next standard* next organi*ation) or (iso near/10 (certif* or "9001" or standard* or system* or qualified or quality or based or assessment*))):ti,ab,kw (Word variations have been searched) | 4154 |
| #15 | "Joint Commission on Accreditation of Healthcare Organizations":ti,ab,kw (Word variations have been searched) | 6 |
| #16 | (#12 or #13 or #14 or #15) | 4189 |
| #17 | (#11 and #16) Publication Year from 2013 to 2014, in Cochrane Reviews (Reviews and Protocols) | 46 |

**Database: Embase 1974 to 2014 June 30**

**Search date: July 1, 2014**

| 1 | cancer center/ or hospice/ or pain clinic/ or rehabilitation center/ or exp hospital/ | 715114 |
| --- | --- | --- |
| 2 | hospital$1.tw. | 1007580 |
| 3 | ward$1.tw. | 56298 |
| 4 | medical clinic$.tw. | 2554 |
| 5 | private clinic$.tw. | 2173 |
| 6 | ((academic or university) adj medical adj (center$ or centre$)).tw. | 18011 |
| 7 | university health facilit$.tw. | 0 |
| 8 | (health facilit$ adj (department$ or unit$)).tw. | 2 |
| 9 | (cancer care adj (facilit$ or unit$)).tw. | 45 |
| 10 | (cancer adj2 (center$ or centre$)).tw. | 21192 |
| 11 | (cancer adj (clinic$ or institute$)).tw. | 20405 |
| 12 | oncology service$.tw. | 1335 |
| 13 | ((oncology or oncologic) adj care unit$).tw. | 6 |
| 14 | (cardiac care adj (facilit$ or unit$)).tw. | 591 |
| 15 | cardiology service$.tw. | 469 |
| 16 | ((coronary or stroke) adj2 unit$).tw. | 9335 |
| 17 | ccu$1.tw. | 3063 |
| 18 | ((cardiologic or cva) adj unit$).tw. | 18 |
| 19 | (heart adj (center$ or centre$)).tw. | 1012 |
| 20 | hospice$.tw. | 11031 |
| 21 | (pain adj (clinic$ or center$ or centre$)).tw. | 4231 |
| 22 | acute pain service$.tw. | 518 |
| 23 | pain relief unit$.tw. | 22 |
| 24 | (rehabilitation adj (center$ or centre$ or clinic$ or department$ or service$ or unit$)).tw. | 14520 |
| 25 | (rehab adj (center$ or centre$)).tw. | 72 |
| 26 | (psychiatric adj (clinic$ or department$ or unit$ or health facilit$)).tw. | 7106 |
| 27 | (mental adj (institution$ or health facilit$)).tw. | 841 |
| 28 | psychiatry unit$.tw. | 495 |
| 29 | day clinic$.tw. | 2110 |
| 30 | (surgicenter$ or surgicentre$).tw. | 112 |
| 31 | ((surgery or surgical) adj (center$ or centre$ or department$)).tw. | 10371 |
| 32 | surgical service$.tw. | 2522 |
| 33 | ((ambulatory or outpatient) adj (surgery or surgical) adj facilit$).tw. | 150 |
| 34 | (geriatric adj (center$ or centre$ or clinic$ or institute$)).tw. | 1011 |
| 35 | (child adj2 clinic$).tw. | 2937 |
| 36 | children institution$.tw. | 49 |
| 37 | (child health adj (center$ or centre$)).tw. | 474 |
| 38 | ((pediatric or paediatric) adj2 (center$ or centre$)).tw. | 5951 |
| 39 | ((pediatric or paediatric) adj (clinic$ or unit$)).tw. | 5150 |
| 40 | ((delivery or delivering or labo?r) adj room$).tw. | 2369 |
| 41 | delivery unit$.tw. | 582 |
| 42 | (maternity adj (clinic$ or home$ or unit$)).tw. | 2856 |
| 43 | midwifery service$.tw. | 208 |
| 44 | ((birth or birthing) adj (center$ or centre$)).tw. | 658 |
| 45 | ((gynecology or obstetrics) adj department$).tw. | 1132 |
| 46 | (obstetric$ adj service$).tw. | 971 |
| 47 | ((operation or operating) adj room$).tw. | 23041 |
| 48 | ((operation or operating or surgical) adj (theater$ or theatre$)).tw. | 5589 |
| 49 | (outpatient adj (department$ or clinic$ or unit$ or service$)).tw. | 37110 |
| 50 | ambulatory care facilit$.tw. | 195 |
| 51 | outdoor clinic$.tw. | 24 |
| 52 | pol#clinic$1.tw. | 4145 |
| 53 | ((radiology or x-ray or radiodiagnosis or radiography or radiological or radiotherapy or roentgen) adj department$).tw. | 4635 |
| 54 | roentgen facilit$.tw. | 3 |
| 55 | radiology service$.tw. | 459 |
| 56 | recovery room$.tw. | 3427 |
| 57 | ((post anesthesia or postanesthesia) adj care unit$).tw. | 1684 |
| 58 | pacu$1.tw. | 2148 |
| 59 | ((emergency or "a & e" or "a&e" or "a and e" or casualty) adj (department$ or ward$)).tw. | 68094 |
| 60 | (("a & e" or "a&e" or "a and e") adj service$).tw. | 66 |
| 61 | (emergency adj (room$ or unit$)).tw. | 20716 |
| 62 | (trauma adj (center$ or centre$ or unit$)).tw. | 11830 |
| 63 | (intensive adj (care or therapy) adj unit$).tw. | 90558 |
| 64 | (icu$1 or itu$1 or picu$1 or pitu$1 or nicu$1 or nitu$1 or itun$1).tw. | 68789 |
| 65 | (burn$1 adj (unit$ or center$ or centre$)).tw. | 4169 |
| 66 | admitting department$.tw. | 48 |
| 67 | (medical record$1 adj (department$ or service$)).tw. | 457 |
| 68 | health information management service$.tw. | 1 |
| 69 | nuclear medicine department$.tw. | 926 |
| 70 | ((hemodialysis or renal dialysis) adj unit$).tw. | 1018 |
| 71 | ((self or minimal or cooperative) adj care unit$).tw. | 49 |
| 72 | observation unit$.tw. | 736 |
| 73 | (pre adj (admission or admitting) adj unit$).tw. | 5 |
| 74 | step down unit$.tw. | 278 |
| 75 | medical assessment unit$.tw. | 141 |
| 76 | (anesthesia adj2 department$).tw. | 475 |
| 77 | anesthesiology service$.tw. | 56 |
| 78 | (occupational therap$ adj (department$ or service$)).tw. | 543 |
| 79 | pathology department$.tw. | 1891 |
| 80 | (physical therap$ adj (department$ or service$)).tw. | 366 |
| 81 | (respiratory therap$ adj (department$ or service$)).tw. | 76 |
| 82 | respiratory care unit$.tw. | 144 |
| 83 | social work department$.tw. | 143 |
| 84 | urology department$.tw. | 824 |
| 85 | venereal disease department$.tw. | 4 |
| 86 | endoscopy department$.tw. | 134 |
| 87 | ((clinical or nuclear) adj pharmacy service$).tw. | 696 |
| 88 | inpatient pharmac$.tw. | 196 |
| 89 | ((intravenous therap$ or iv therapy) adj department$).tw. | 6 |
| 90 | nursing unit$.tw. | 1480 |
| 91 | or/1-90 | 1556726 |
| 92 | accreditation/ or certification/ | 45687 |
| 93 | accredit$.tw. | 17787 |
| 94 | (jcaho or jcia or urac or equip or carf).tw. | 3078 |
| 95 | "Joint Commission on Accreditation of Healthcare Organizations".tw. | 802 |
| 96 | "evaluation and quality improvement program".tw. | 8 |
| 97 | (international organi#ation adj2 standard$).tw. | 790 |
| 98 | (international standard$ adj organi#ation).tw. | 364 |
| 99 | (iso adj10 (certif$ or "9001" or standard$ or system$ or qualified or quality or based or assessment$)).tw. | 5281 |
| 100 | or/92-99 | 61341 |
| 101 | 91 and 100 | 13201 |
| 102 | limit 101 to "reviews (best balance of sensitivity and specificity)" | 1436 |
| 103 | 2013$.em,dp,dd,yr. | 1836794 |
| 104 | 2014$.em,dp,dd,yr. | 895484 |
| 105 | 103 or 104 | 2395763 |
| 106 | 102 and 105 | 159 |
| 107 | clinical trial/ | 836308 |
| 108 | randomized controlled trial/ | 347009 |
| 109 | randomization/ | 62452 |
| 110 | double blind procedure/ | 116447 |
| 111 | single blind procedure/ | 18444 |
| 112 | crossover procedure/ | 39305 |
| 113 | placebo/ | 254263 |
| 114 | placebo$.tw. | 203743 |
| 115 | randomi?ed controlled trial$.tw. | 99958 |
| 116 | rct.tw. | 14160 |
| 117 | random allocation.tw. | 1356 |
| 118 | randomly allocated.tw. | 20559 |
| 119 | allocated randomly.tw. | 1950 |
| 120 | (allocated adj2 random).tw. | 793 |
| 121 | single blind$.tw. | 14591 |
| 122 | double blind$.tw. | 147546 |
| 123 | ((treble or triple) adj blind$).tw. | 397 |
| 124 | prospective study/ | 254413 |
| 125 | or/107-124 | 1383778 |
| 126 | case study/ | 26663 |
| 127 | case report.tw. | 268762 |
| 128 | abstract report/ | 89615 |
| 129 | letter/ | 822724 |
| 130 | human/ | 14754086 |
| 131 | nonhuman/ | 4325715 |
| 132 | animal/ | 1569539 |
| 133 | animal experiment/ | 1782696 |
| 134 | 131 or 132 or 133 | 6218654 |
| 135 | 134 not (130 and 134) | 4947706 |
| 136 | or/126-129,135 | 6067526 |
| 137 | 125 not 136 | 1295767 |
| 138 | 101 and 137 | 372 |
| 139 | 105 and 138 | 74 |
| 140 | evaluation.sh. | 170349 |
| 141 | evaluation stud$.tw. | 4418 |
| 142 | "types of study".sh. | 630 |
| 143 | intervention study.sh. | 19973 |
| 144 | (intervention$ adj (stud$ or trial$)).tw. | 26934 |
| 145 | comparative study.sh. | 635513 |
| 146 | comparative stud$.tw. | 91244 |
| 147 | experimental study.sh. | 14139 |
| 148 | experimental stud$.tw. | 82789 |
| 149 | (time adj series).tw. | 18106 |
| 150 | (pre test or pretest or post test or posttest).tw. | 20997 |
| 151 | or/140-150 | 998611 |
| 152 | 101 and 151 | 615 |
| 153 | 105 and 152 | 79 |
| 154 | 106 or 139 or 153 | 293 |

**Database: MEDLINE(R) In-Process & Other Non-Indexed Citations, Ovid MEDLINE(R) Daily, Ovid MEDLINE(R) and Ovid OLDMEDLINE(R) 1946 to Present**

**Search date: July 1, 2014**

| 1 | exp hospital departments/ or exp hospital units/ or exp hospitals/ or rehabilitation centers/ | 400826 |
| --- | --- | --- |
| 2 | hospital$1.tw. | 744252 |
| 3 | ward$1.tw. | 40744 |
| 4 | medical clinic$.tw. | 2083 |
| 5 | private clinic$.tw. | 1586 |
| 6 | ((academic or university) adj medical adj (center$ or centre$)).tw. | 13990 |
| 7 | university health facilit$.tw. | 0 |
| 8 | (health facilit$ adj (department$ or unit$)).tw. | 2 |
| 9 | (cancer care adj (facilit$ or unit$)).tw. | 24 |
| 10 | (cancer adj2 (center$ or centre$)).tw. | 13119 |
| 11 | (cancer adj (clinic$ or institute$)).tw. | 14698 |
| 12 | oncology service$.tw. | 790 |
| 13 | ((oncology or oncologic) adj care unit$).tw. | 4 |
| 14 | (cardiac care adj (facilit$ or unit$)).tw. | 408 |
| 15 | cardiology service$.tw. | 293 |
| 16 | ((coronary or stroke) adj2 unit$).tw. | 6360 |
| 17 | ccu$1.tw. | 2454 |
| 18 | ((cardiologic or cva) adj unit$).tw. | 11 |
| 19 | (heart adj (center$ or centre$)).tw. | 533 |
| 20 | hospice$.tw. | 8398 |
| 21 | (pain adj (clinic$ or center$ or centre$)).tw. | 2707 |
| 22 | acute pain service$.tw. | 303 |
| 23 | pain relief unit$.tw. | 18 |
| 24 | (rehabilitation adj (center$ or centre$ or clinic$ or department$ or service$ or unit$)).tw. | 9801 |
| 25 | (rehab adj (center$ or centre$)).tw. | 39 |
| 26 | (psychiatric adj (clinic$ or department$ or unit$ or health facilit$)).tw. | 5079 |
| 27 | (mental adj (institution$ or health facilit$)).tw. | 664 |
| 28 | psychiatry unit$.tw. | 327 |
| 29 | day clinic$.tw. | 1401 |
| 30 | (surgicenter$ or surgicentre$).tw. | 95 |
| 31 | ((surgery or surgical) adj (center$ or centre$ or department$)).tw. | 7637 |
| 32 | surgical service$.tw. | 2141 |
| 33 | ((ambulatory or outpatient) adj (surgery or surgical) adj facilit$).tw. | 127 |
| 34 | (geriatric adj (center$ or centre$ or clinic$ or institute$)).tw. | 670 |
| 35 | (child adj2 clinic$).tw. | 2371 |
| 36 | children institution$.tw. | 37 |
| 37 | (child health adj (center$ or centre$)).tw. | 464 |
| 38 | ((pediatric or paediatric) adj2 (center$ or centre$)).tw. | 3860 |
| 39 | ((pediatric or paediatric) adj (clinic$ or unit$)).tw. | 3834 |
| 40 | ((delivery or delivering or labo?r) adj room$).tw. | 1753 |
| 41 | delivery unit$.tw. | 471 |
| 42 | (maternity adj (clinic$ or home$ or unit$)).tw. | 2362 |
| 43 | midwifery service$.tw. | 214 |
| 44 | ((birth or birthing) adj (center$ or centre$)).tw. | 585 |
| 45 | ((gynecology or obstetrics) adj department$).tw. | 762 |
| 46 | (obstetric$ adj service$).tw. | 891 |
| 47 | ((operation or operating) adj room$).tw. | 18082 |
| 48 | ((operation or operating or surgical) adj (theater$ or theatre$)).tw. | 4059 |
| 49 | (outpatient adj (department$ or clinic$ or unit$ or service$)).tw. | 25312 |
| 50 | ambulatory care facilit$.tw. | 160 |
| 51 | outdoor clinic$.tw. | 13 |
| 52 | pol#clinic$1.tw. | 3291 |
| 53 | ((radiology or x-ray or radiodiagnosis or radiography or radiological or radiotherapy or roentgen) adj department$).tw. | 3385 |
| 54 | roentgen facilit$.tw. | 3 |
| 55 | radiology service$.tw. | 349 |
| 56 | recovery room$.tw. | 2655 |
| 57 | ((post anesthesia or postanesthesia) adj care unit$).tw. | 1439 |
| 58 | pacu$1.tw. | 1473 |
| 59 | ((emergency or "a & e" or "a&e" or "a and e" or casualty) adj department$).tw. | 49650 |
| 60 | (("a & e" or "a&e" or "a and e") adj service$).tw. | 63 |
| 61 | (emergency adj (room$ or unit$)).tw. | 13871 |
| 62 | (trauma adj (center$ or centre$ or unit$)).tw. | 9806 |
| 63 | (intensive adj (care or therapy) adj unit$).tw. | 68976 |
| 64 | (icu$1 or itu$1 or picu$1 or pitu$1 or nicu$1 or nitu$1 or itun$1).tw. | 39241 |
| 65 | (burn$1 adj (unit$ or center$ or centre$)).tw. | 3201 |
| 66 | admitting department$.tw. | 32 |
| 67 | (medical record$1 adj (department$ or service$)).tw. | 351 |
| 68 | health information management service$.tw. | 2 |
| 69 | nuclear medicine department$.tw. | 507 |
| 70 | ((hemodialysis or renal dialysis) adj unit$).tw. | 800 |
| 71 | ((self or minimal or cooperative) adj care unit$).tw. | 45 |
| 72 | observation unit$.tw. | 512 |
| 73 | (pre adj (admission or admitting) adj unit$).tw. | 0 |
| 74 | step down unit$.tw. | 163 |
| 75 | medical assessment unit$.tw. | 74 |
| 76 | (anesthesia adj2 department$).tw. | 341 |
| 77 | anesthesiology service$.tw. | 38 |
| 78 | (occupational therap$ adj (department$ or service$)).tw. | 393 |
| 79 | pathology department$.tw. | 1250 |
| 80 | (physical therap$ adj (department$ or service$)).tw. | 316 |
| 81 | (respiratory therap$ adj (department$ or service$)).tw. | 67 |
| 82 | respiratory care unit$.tw. | 108 |
| 83 | social work department$.tw. | 109 |
| 84 | urology department$.tw. | 522 |
| 85 | venereal disease department$.tw. | 6 |
| 86 | endoscopy department$.tw. | 82 |
| 87 | ((clinical or nuclear) adj pharmacy service$).tw. | 461 |
| 88 | inpatient pharmac$.tw. | 109 |
| 89 | ((intravenous therap$ or iv therapy) adj department$).tw. | 2 |
| 90 | nursing unit$.tw. | 1193 |
| 91 | or/1-90 | 1146483 |
| 92 | exp accreditation/ or certification/ | 28588 |
| 93 | accredit$.tw. | 14019 |
| 94 | (jcaho or jcia or urac or equip or carf).tw. | 2547 |
| 95 | "Joint Commission on Accreditation of Healthcare Organizations".tw. | 740 |
| 96 | "evaluation and quality improvement program".tw. | 8 |
| 97 | (international organi#ation adj2 standard$).tw. | 671 |
| 98 | (international standard$ adj organi#ation).tw. | 284 |
| 99 | (iso adj10 (certif$ or "9001" or standard$ or system$ or qualified or quality or based or assessment$)).tw. | 3810 |
| 100 | or/92-99 | 41875 |
| 101 | 91 and 100 | 9486 |
| 102 | limit 101 to "reviews (best balance of sensitivity and specificity)" | 595 |
| 103 | 2013$.ed,ep,yr,dp. | 1567767 |
| 104 | 2014$.ed,ep,yr,dp. | 888351 |
| 105 | 103 or 104 | 1982384 |
| 106 | 102 and 105 | 58 |
| 107 | randomized controlled trial.pt. | 377177 |
| 108 | controlled clinical trial.pt. | 88615 |
| 109 | randomi#ed.ab,ti. | 380991 |
| 110 | placebo.ab,ti. | 159646 |
| 111 | clinical trials as topic.sh. | 170604 |
| 112 | randomly.ab,ti. | 215836 |
| 113 | trial.ti,ab. | 367009 |
| 114 | or/107-113 | 1044728 |
| 115 | exp animals/ not humans.sh. | 3960142 |
| 116 | 114 not 115 | 958369 |
| 117 | 101 and 116 | 187 |
| 118 | 105 and 117 | 35 |
| 119 | evaluation studies.pt,sh. | 195265 |
| 120 | evaluation stud$.tw. | 3532 |
| 121 | intervention studies.sh. | 6789 |
| 122 | intervention stud$.tw. | 11726 |
| 123 | comparative study.pt,sh. | 1682478 |
| 124 | comparative stud$.tw. | 76676 |
| 125 | experimental stud$.tw. | 75192 |
| 126 | (time adj series).tw. | 16128 |
| 127 | (pre test or pretest or post test or posttest).tw. | 16133 |
| 128 | or/119-127 | 1973100 |
| 129 | 101 and 128 | 582 |
| 130 | 105 and 129 | 66 |
| 131 | 106 or 118 or 130 | 151 |
